# Using an Electronic App to Promote Home-Based Self-Care in Older Patients With Heart Failure: Qualitative Study on Patient and Informal Caregiver Challenges

**DOI:** 10.2196/15885

**Published:** 2020-11-09

**Authors:** Sahr Wali, Karim Keshavjee, Linda Nguyen, Lawrence Mbuagbaw, Catherine Demers

**Affiliations:** 1 Centre for Global eHealth Innovation Techna Institute University Health Network Toronto, ON Canada; 2 Institute of Health Policy, Management and Evaluation Dalla Lana School of Public Health University of Toronto Toronto, ON Canada; 3 InfoClin Toronto, ON Canada; 4 School of Rehabilitation Science Faculty of Health Sciences McMaster University Hamilton, ON Canada; 5 Department of Health Research Methods, Evidence and Impact McMaster University Hamilton, ON Canada; 6 Department of Medicine McMaster University Hamilton, ON Canada

**Keywords:** mobile health, mobile apps, heart failure, self-care, mobile phone

## Abstract

**Background:**

Heart failure (HF) affects many older individuals in North America, with recurrent hospitalizations despite postdischarge strategies to prevent readmission. Proper HF self-care can potentially lead to better clinical outcomes, yet many older patients find self-care challenging. Mobile health (mHealth) apps can provide support to patients with respect to HF self-care. However, many mHealth apps are not designed to consider potential patient barriers, such as literacy, numeracy, and cognitive impairment, leading to challenges for older patients. We previously demonstrated that a paper-based standardized diuretic decision support tool (SDDST) with daily weights and adjustment of diuretic dose led to improved self-care.

**Objective:**

The aim of this study is to better understand the self-care challenges that older patients with HF and their informal care providers (CPs) face on a daily basis, leading to the conversion of the SDDST into a user-centered mHealth app.

**Methods:**

We recruited 14 patients (male: 8/14, 57%) with a confirmed diagnosis of HF, aged ≥60 years, and 7 CPs from the HF clinic and the cardiology ward at the Hamilton General Hospital. Patients were categorized into 3 groups based on the self-care heart failure index: patients with adequate self-care, patients with inadequate self-care without a CP, or patients with inadequate self-care with a CP. We conducted semistructured interviews with patients and their CPs using persona-scenarios. Interviews were transcribed verbatim and analyzed for emerging themes using an inductive approach.

**Results:**

Six themes were identified: usability of technology, communication, app customization, complexity of self-care, usefulness of HF-related information, and long-term use and cost. Many of the challenges patients and CPs reported involved their unfamiliarity with technology and the lack of incentive for its use. However, participants were supportive and more likely to actively use the HF app when informed of the intervention’s inclusion of volunteer and nurse assistance.

**Conclusions:**

Patients with varying self-care adequacy levels were willing to use an mHealth app if it was simple in its functionality and user interface. To promote the adoption and usability of these tools, patients confirmed the need for researchers to engage with end users before developing an app. Findings from this study can be used to help inform the design of an mHealth app to ensure that it is adapted for the needs of older individuals with HF.

## Introduction

### Burden of Disease

Heart failure (HF) has been defined as a global epidemic affecting 26 million individuals worldwide [1]. With the increasing aging population, HF is the leading cause of hospitalization and mortality in older adults, placing a significant clinical and financial burden on the health care system [2,3]. Patients with HF currently have longer hospital stays, and up to 50% of them are readmitted within 3 months post discharge [3,4]. HF-related readmission is attributed to worsening symptoms and clinical deterioration [5-7]. However, studies have found that the adoption of self-care in patients with HF could lead to a reduction of more than 30% of HF-related hospital readmissions [5,6].

### Importance of Self-Care

Self-care is a decision-making process that involves the choice of various behaviors to maintain physiological stability in the face of disease and the appropriate response to symptoms when they occur [7,8]. The process of HF self-care comprises of 3 separate but connected components: (1) self-maintenance, (2) symptom perception, and (3) self-management. Self-maintenance consists of actions associated with treatment adherence [8]. Symptom perception involves individual detection, assurance, and interpretation of physical sensations (ie, body listening and labeling of symptoms). Self-management involves the response to changes in symptoms [8]. Each component of the HF self-care process represents key tasks pivotal to HF stability. Self-management is often inferred as the major area of focus to improve HF outcomes, as it is directly associated with the response to changes in symptoms [8]. However, literature has found that all 3 self-care constructs reflect processes that build on one another and move in sequence to maintain, recognize, and manage physiological stability [8].

Proper HF self-care involves a series of tasks such as daily weight and symptom monitoring and adjusting diuretics based on the patient’s symptoms [9-12]. Weight monitoring is identified as a pivotal component of HF self-care, as weight gain is the last common step before worsening clinical outcomes [13]. In the Acute Study of Clinical Effectiveness of Nesiritide in Decompensated Heart Failure randomized control trial (RCT), an increase in body weight after hospitalization was independently associated with a 16% increase (per kg) in the likelihood of 30-day mortality or hospital readmission (hazard ratio per kg increase 1.16; 95% CI 1.09-1.23; *P*<.001) [13]. To effectively manage weight fluctuations and reduce fluid overload, as part of HF self-care, patients should be empowered to manage and adjust their own diuretics in the home setting [11].

Despite the benefits associated with HF self-care, many older adults find the process of self-care challenging [14,15]. Factors such as the absence of an informal care provider (CP; ie, spouse, family member, and friend), poor economic stability, presence of comorbidities, limited knowledge about HF self-care, and the presence of cognitive impairment can potentially limit patients’ ability to properly manage their symptoms [9,10,14-17]. Many studies have also reported that patient values are integral to how patients respond to the severity of their symptoms; thus, poor patient adherence could be related to a lack of perceived need or motivation for self-care [17]. Ultimately, to better support patients with their self-care challenges, we must understand the barriers and facilitators they face, to address their unmet needs.

### Leveraging Technology to Support Self-Care

With the increasing popularity of mobile phones, the use of mobile health (mHealth) apps can potentially support the process of self-care [2,18,19]. In a systematic review evaluating the state of mHealth apps available for cardiovascular disease, including HF, they found that patients using mHealth apps had greater treatment adherence compared with usual care (odds ratio 4.51, 95% CI 2.38-8.57; *P*<.001) [2]. However, older adults with HF have complex needs, leaving many older adults to not commonly use mHealth apps because of the perception that they are not suited to their needs or capabilities [2,18-20]. Specifically, many older adults with HF have low levels of health and computer literacy, mild cognitive impairment, and visual and hearing challenges, all of which contribute to their poor use of technology [19,21,22]. To improve the adoption of these tools among older adults, mHealth apps should be created using a more user-centered design (UCD) approach to address their needs and limitations [19,23].

We previously conducted a pilot RCT (ClinicalTrials.gov Identifier: NCT01886534) that tested the use of a standardized diuretic decision tool (SDDST; Registered Copyright: 1105713) combined with a talking weight scale, nursing support with home visits, and a literacy and numeracy-sensitive information booklet. The results of this RCT demonstrated that self-management improved significantly in the intervention arm compared with usual care (*P*=.005) [24]. The intervention was safe and feasible. The objective of this study is to better understand the perspectives of patients and care providers (CPs) on their self-care challenges to incorporate their lived context into the design of an HF app (HFApp). Ultimately, the information collected will allow us to convert the SDDST into a user-centered mHealth app to better support HF self-care.

## Methods

### Study Design

This qualitative descriptive study was guided by the evidence-based UCD framework [25,26]. The study focused on the first phase of the framework to identify end users’ needs through a series of semistructured interviews with older patients with HF and their CPs. This approach allowed us to understand the perspectives of patients and CPs, which will inform the design of the electronic version of the HFApp. This study was approved by the Hamilton Integrated Research Ethics Board.

### Study Population

A convenience sample of patients at Hamilton General Hospital was invited to participate in the study via telephone (SW). The study population included males and females aged ≥60 years with a primary diagnosis of HF. Both patients admitted to the hospital with a primary diagnosis of HF and patients followed in the HF clinic were considered, as it allowed us to obtain a broad representation of HF diagnoses. The following patients were excluded: (1) those who resided in a long-term care facility, (2) those whose life expectancy was <3 months, (3) those who were referred for cardiovascular surgery before hospital discharge, (4) those who were not on a loop diuretic by mouth, (5) those who were currently on dialysis, or (6) those who were unable to speak English.

Informal caregivers (males and females aged ≥18 years) eligible for recruitment in this study were required to provide the patient with at least 4 hours of ongoing patient support a week (ie, spouse, family member, and friend). CPs were only approached for study participation once patient telephone confirmation was received.

### Participant Categorization

We based the UCD framework on the foundation that just as users differ in their technology adequacy levels, patients with HF differ in their levels of self-care adequacy as well [25]. Thus, to ensure that patients from varying self-care adequacy levels were included in this study, we used the validated self-care heart failure index (SCHFI) to appropriately categorize patients [11]. Patients were categorized into 3 different groups according to the presence of a CP and their level of self-care adequacy, where an average score of 70 or higher on the SCHFI was labeled as self-care adequate: (1) adequate self-care with a CP, (2) inadequate self-care without a CP, and (3) inadequate self-care with a CP [11].

All CPs were categorized into one participant group and completed the caregiver contribution self-care heart failure index (CC-SCHFI) [27]. The CC-SCHFI is a modification of the SCHFI, with the same scales for self-care maintenance, self-care management, and self-care confidence. However, the use of the CC-SCHFI allowed for the CPs’ contribution to the patients’ HF self-care to be measured specific to the 3 main areas of self-care [27]. These scores were also used to evaluate differences in CC-SCHFI scores and CP interview feedback.

### Sample Size

We planned to approach a maximum of 20 individuals to participate in the study, consisting of 15 patients with HF (5 within each patient group) and 5 CPs, or until data saturation was reached. We used the guideline of Malterud et al [28] to estimate when data saturation would be reached for both patients and CPs. Due to the study aim and direct participant dialog, a sample size of 20 participants was needed to achieve data saturation.

### Patient and Caregiver Interviews

Patients and their CPs participated in one individual semistructured interview. Interviews were held separately for each patient at the Hamilton General Hospital for approximately 2 hours. For patient and CP convenience, interviews were held together with the patient and the CP. Before the start of the interviews, patients and CPs signed an informed consent form. The same individual (SW) conducted all interview sessions using an interview guide script to ensure consistency.

Following participant consent, the interview facilitator (SW) provided a detailed overview of the main components of the interview. Each participant was given a tailored discussion guide containing a summary of the HFApp intervention, a series of mock-ups to visualize the HFApp, and a list of persona-scenarios for discussion (Multimedia Appendices 1 and 2) [29]. Persona-scenarios are documents that have commonly been used in UCD studies to help represent a type of individual that the participant can relate with [30]. A persona describes different types of users, or patients, based on their goals and key behaviors (ie, difficulties adjusting diuretics or using technology). The scenario describes specific situations that the persona may face and subsequently impact their care (ie, forgetting to use the app or visiting relatives). A persona-scenario is commonly used to help provide insight into the patient’s needs and expectations, as it allows them to draw on information from another’s experience and compare it with their own [7,29].

During the interview, each participant was asked to review one of the listed personas and evaluate whether the HFApp would be an effective tool with respect to the different scenarios. Each participant was also asked to come up with at least one idea on how to improve the intervention. Feedback and suggestions were audio-recorded and transcribed verbatim for analysis.

### Data Analysis

Interviews were transcribed verbatim using Microsoft Word and imported into NVivo, Version 10 (QSR International), for data analysis. Braun and Clarke’s [31] inductive thematic analysis approach was used to analyze, identify, reflect, and refine emerging themes from the interviews. Two researchers (SW and LN) reviewed the design themes independently (investigator triangulation). The research team was also debriefed about the resultant design themes to ensure that multiple perspectives were incorporated during analysis. The interviewer (SW) was the primary investigator identifying the codes, categories, and themes for the data analysis.

Following thematic analysis, feedback from participant interviews was used to evaluate the changes needed within the HFApp intervention. A series of actions and items corresponding to each theme was developed using Braun and Clarke’s [31] methods for qualitative research.

## Results

### Participant Characteristics

A total of 21 participant interviews were conducted among 14 patients (8/14, 7% male) and 7 CPs (3/7, 43% male). Within the patient groups, there were 6 patients categorized as adequate self-care patients, 4 patients as inadequate self-care patients with a CP, and 4 patients as inadequate self-care patients without a CP. The patients had a mean age of 74 (SD 4) years and a mean left ventricular ejection fraction of 32% (SD 16%). The CPs had an average age of 66 (SD 16) years.

### Discussion Sessions

A total of 6 themes were identified: (1) usability of technology, (2) communication, (3) app customization, (4) complexity of self-care, (5) usefulness of HF-related information, and (6) cost and long-term use. The results are summarized in Table 1 and further described in the following section under each design theme. Feedback from each patient with HF is denoted by *P*, whereas each CP is denoted by *C*.

**Table 1:** Design themes derived from patient and care provider interviews.

Design themes	Factors/design requirement
1. Usability of technology	Perception that technology will make self-care more challenging Incentive for technology use needed Willingness to use technology if kept simple
2. Communication	Use of direct communication (in person and virtual) with nurse highly desired Open sharing and access to patient information to improve communication
3. App customization	Management of medications on one device Addition of notifications at patient’s desired time/manner Customization of audio and visual format for each patient during setup
4. Complexity of self-care	Perception that daily management of HF^a^ self-care is difficult Difficulty with diuretic adjustment Benefits of nursing support
5. Usefulness of HF-related information	Provision of information from physician and nurses difficult to understand Interest in information relevant to specific patients
6. Long-term use and costs	Concerns with potential dependence on the HFApp intervention and future costs Integration with current device for long-term use and reduce cost

^a^HF: heart failure.

### Design Theme 1: Usability of Technology

#### Perception That Technology Will Make Self-Care More Challenging

Many participants expressed significant resistance regarding the use of technology. When patients and CPs were asked about their current technology usage, they indicated that they either did not use them or specifically did not use any device for HF self-care because of the perception that it would only create more challenges for them:

I don’t like using any technology, it just makes more problems.

P, inadequate with CP

With the consistent negative perception around the use of technology, many patients assumed that the HFApp would become another barrier to their self-care, before the intervention was even fully described. They felt that they were already unfamiliar with technology; therefore, its addition would only further complicate their self-care regimen. Patients and CPs also indicated that they preferred to consult with a real person to manage their HF, as it gave them a sense of comfort and added a *human-touch* to their care. They felt that they already had limited contact with their physicians; thus, the addition of technology would only further isolate their care and contribute to their self-care challenges:

I think he would have a lot of difficulty learning how to use it. He barely knows how to use his phone.

C, adequate

#### Lack of Incentive for Technology Use

Both patients and CPs mentioned how they did not view the use of technology as an added benefit to their current treatment regimen, as they would be completing the same tasks with or without it. Due to past difficulties with technology, patients and CPs did not see the need or motivation to use a new device, as they associated the technology as another barrier to their self-care adequacy, rather than a beneficial tool to mitigate some of their self-care challenges:

If I’m already writing out my weight everyday and doing fine, I don’t see a reason for me to stop what I’m doing and learn something new, like what’s the point?

P, adequate with CP

#### Willingness to Use Technology If Kept Simple

After the full HFApp intervention was explained, patients and CPs had a better evaluation of their potential technology usage. They indicated that if the app was simple, as displayed in the mock-ups (Multimedia Appendix 2), they would be willing to learn how to use it. During the persona-scenario discussion, many patients indicated that they shared the same frustrations as the persona *Diane Lambert*, who was unfamiliar with iPads and tablets (Multimedia Appendix 1). They described how learning to use the HFApp would be easier if there were only a few functionalities:

I think the app would help Diane (persona), but only if it was really simple. A lot of apps have too many things going on, so I get lost.

P, adequate with CP

### Design Theme 2: Communication

#### Use of Direct Communication (In Person and Over Phone) With Nurse Highly Desired

Most patients and CPs articulated how one of their major difficulties involved being able to contact their physician or nurse for support when they were at home. To help resolve this challenge, patients stressed that having a direct source of communication with a member of their care team would be highly beneficial. Specifically, patients and CPs indicated that if the HFApp had nursing support dedicated to answering calls for the intervention, this would help improve both the quality and reliability of the HFApp support as a whole:

My doctor even gave me his cell phone number, but I still can’t reach him.

P, inadequate without CP

During the persona-scenario discussion, patients and CPs also added how being able to receive additional information from a nurse would help them become more confident in their self-care decision making, similar to the persona Christina Williams (Multimedia Appendix 1). They would not have to rely solely on the technology of the HFApp for guidance, which made the use of the entire HF self-care intervention more comforting.

#### Open Sharing and Access to Patient Information to Improve Communication

Participants emphasized how if the intervention was able to provide nurses and physicians access to their patient’s HFApp information, with consent, this would allow for greater accuracy during assessments. This aspect of the HFApp was strongly appealing as patients often felt that there was a gap in their quality of care because their health care provider did not understand their current health condition:

The worst is when they think [they are] right, but they don’t understand that my symptoms are not the same as before.

P, adequate with CP

Patients added that in many cases, they could not remember the depth of their medical history from their last clinic visit, making it difficult for them to reexplain or update the physician or nurse with their health status. They often found it difficult to provide accurate information, as they were not able to remember all of their symptoms and did not always record their weight. Patients and CPs highlighted that by providing physicians and nurses with access to patient information collected by the HFApp, this would help update them during in-person consults and over phone support calls:

I try to record my weight, but when my cardiologist asks me questions I don’t know what to say...I think having the app track it would be really good for me cause I get lazy.

P, inadequate with CP

Information recorded within the HFApp was also deemed beneficial for patients who were able to adequately perform self-care, as they would be able to describe how their HF symptoms worsened even when they were adherent to the process of self-care.

### Design Theme 3: App Customization

When participants were asked about using the HFApp, the concept of customization was a major factor that influenced their decision to use the app for self-care. Patients mentioned how the ability to tailor the tool to their needs would increase the overall appeal and usability of the app, as older adults with HF have varying needs and capabilities.

#### Management of Medications on One Device

Patients were disappointed with the inability to manage multiple medications within the design of the current HFApp. Some patients stressed that they would become reliant on the technology and would need one common system to manage all their medications. As patients with HF are often required to take many medications, they believed that being able to track all of them in one place would make their self-care easier:

I have diabetes too, so why wouldn’t I be able to manage both? I could have like a separate space for it...I think it would be really helpful.

P, adequate with CP

#### Addition of Notifications at Patient’s Desired Time/Manner

Patients and CPs began to acknowledge the benefits of using the HFApp; however, they stressed that there was a need for notifications and reminders to be integrated within the HFApp to obtain its optimal functionality. They stated that it is often difficult to maintain their treatment routine because of a number of factors (eg, tiring, confusing, and limited mobility) but mainly because they are forgetful. To combat this issue, creating a reminder system within the HFApp and setting them according to the patient’s daily routine would help promote treatment adherence:

I always forget to do it. If you don’t tell me I won’t do it. So, if you want me to use this thing, you better buzz me until I do it...For me, I would make sure it kept buzzing me until I got onto that scale.

P, inadequate without CP

#### Customization of Audio and Visual Format for Each Individual Patient During Setup

Many older adults with HF may have visual or hearing impairment. To optimize the app potential, participants suggested that visual and hearing preferences should be adjustable to help accommodate various patient needs and capabilities. Specifically, participants highlighted that during the on-boarding and setup of the HFApp, these customizations can be discussed and assorted with the assistance of a nurse:

I know my dad’s vision is getting worse. He’s too stubborn to admit it, but I think maybe if the app could repeat each thing out loud when you click it that would really help. Or just use really big fonts and bright colors.

C, adequate

### Design Theme 4: Complexity of Self-Care

#### Perception That Daily Management of HF Self-Care Is Difficult

During the persona-scenario discussions, participants had varying attitudes (positive and negative) regarding HF self-care; however, the majority of patients with HF agreed that they found the process to be difficult. They specifically indicated that the daily management was overwhelming, especially when patients had multiple comorbidities:

There’s too many things to remember and I have diabetes, so I mix those up too.

P, inadequate with CP

They viewed the management of HF as a burden for their daily routine. Patients even indicated that they consciously decided not to perform self-care tasks because they felt if they were unable to adequately perform all the tasks, these tasks would not make a difference on their health. In reflection to this, according to the patient SCHFI scores recorded, only 50% (7/14) of the patients interviewed indicated that they weighed themselves on a regular basis.

#### Difficulty With Diuretic Adjustment

Patients reported that they often had difficulty with adjusting their diuretic dosing. Both patients and CPs expressed their fear in changing the dosage incorrectly and potentially worsening their symptoms. They acknowledged the importance of correct diuretic dosing, but their limited self-efficacy highlighted the potential for the HFApp to improve their confidence in completing this task:

My wife asks me to help her, but I don’t know if I’m doing it right either. Every morning I hope her weight is the same, so I don’t have to think about it again.

C, adequate

#### Benefits of Nursing Support

Participants had preestablished views on the complexity of self-care and their difficulty in managing their symptoms. When the inclusion of the nurse home visits, as part of the HFApp intervention, was explained, both patients with and without earlier experience with nurse home visits agreed on the benefits of their presence. The significance of the nursing support varied among patients with inadequate and adequate self-care scores, where patients with inadequate scores felt a stronger need for them compared with patients with adequate self-care scores:

Yeah, I would love that. Just to come and make sure I’m alright...This beats having to wait for an appointment.

P, inadequate with CP

### Design Theme 5: Usefulness of HF-Related Information

#### Provision of Information From Physicians and Nurses Difficult to Understand

Participants expressed the lack of clarity in the information provided to them by both nurses and physicians. Patients specifically expressed how they either did not understand or would simply forget about the information after their appointment:

They keep talking and repeating stuff, but I don’t understand...I just nod my head because I don’t want to disappoint them.

P, inadequate with CP

#### Interest in Information Relevant to Specific Patient

Both CPs and patients felt that they were consistently given generic information regarding their HF self-care. CPs were concerned about this issue, as they felt that the advice from their physician should be held at a higher degree and tailored to their individual case:

My husband is good with managing his weight. He still gets short of breath. I don’t know how to you know help, but I told his doctor, and they don’t seem to get him either.

C, adequate

When CPs and patients reviewed the persona-scenario of *Christina Williams* (Multimedia Appendix 1), a few adequate self-care patients connected with her situation. They agreed on the frustration of following their regimen but still experiencing worsening symptoms. Nonetheless, they identified that if the HFApp could provide specific information relevant to the patient, their physician could use this information as a reference point of discussion during their appointments:

I’m like Christina (persona)… what if I could have my doctor use this info on the app when he talks to me. So he has a better idea of what’s going on.

P, adequate with CP

### Design Theme 6: Long-Term Use and Costs

#### Concerns With Potential Dependence on HFApp Intervention and Future Costs

Following the HFApp explanation, participants were intrigued with the intervention’s implementation; however, they also had concerns about the longevity and sustainability of its use. The HFApp was described as a service free of charge for the patient; however, patients and CPs expressed their fear of potentially becoming dependent on its use and then having to pay for the use of the app later:

I think we need something to tell us that hey you won’t be charged later, and if you are you get a refund or something.

P, inadequate with CP

#### Integration With Current Device for Long-Term Use and Reduce Cost

Some participants recommended integrating the app on current devices (tablet, smartphone, and iPad). They felt that this could reduce the cost for the stakeholder/funder and could improve the usability of the app, as it would allow for a seamless integration with the devices they are currently using:

I already have one of those iPad things, so I don’t really need another one. I can just put your app on my iPad. This way you don’t have to pay or I don’t have to pay now or later, whatever you decide...Either way more bang for your buck.

P, inadequate with CP

### Identifying What’s Next—Summarized Actions and Items

To determine the changes needed for the HFApp intervention, a table outlining each design theme factor with the corresponding action and item was created (Multimedia Appendix 3). The specifics relating to each design theme and the resultant action and item are further described in Multimedia Appendix 3.

## Discussion

### Principal Findings

Our study demonstrates that older adults with HF and their CPs are willing to use an mHealth app to assist them with their self-care. We identified 6 major design themes that provided insight into the challenges associated with patient self-care and the implications it may have for the HFApp. These findings can be translated into app design specifications to improve the usability of the HFApp intervention, as aligned with our study objective. However, older adults have varying complex needs, which will require additional mechanisms of customization within the HFApp to ensure that it is simple, effective, and usable (Multimedia Appendix 3).

Through this study, it became evident that participants had varying experiences with using technology, but patients and CPs commonly felt that it would create more challenges than benefits. Their unfamiliarity with technology made it clear that there was a lack of incentive for using the HFApp intervention, as any source of technology was seen as burdensome. We found this challenge to be common among many older adult populations, as a systematic review of mHealth-based HF interventions similarly found that over 20% of patients failed to even start the use of the mHealth tool because of difficulties with using their mobile phone [32]. They also reported that they had a 60% attrition rate mainly because of patient-reported technical difficulties (ie, complex language and poor user interface) [32]. To combat this issue, participants indicated that the mHealth tool would need to be simple, and they would need to be provided with proper support to ensure that they are comfortable with its use. To facilitate these design requirements, we outlined the following key items to improve the usability of the HFApp: (1) simple instruction manual on HFApp, (2) summary on the benefits of HFApp specific to self-care challenges, (3) refined user interface for older adult needs, and (4) volunteer support for technical issues (Multimedia Appendix 3). These action items were chosen on the basis that the nursing and volunteer technical support were the components that patients and CPs indicated would improve their comfort and confidence with the HFApp [33,34]. This feedback is consistent with other studies that have incorporated the use of nursing or trained volunteer support [35]. In an RCT (n=316) evaluating the use of trained community volunteers to facilitate the uptake of a mHealth intervention (TAPApp) to support self-care in patients aged >70 years, patients indicated that the trained volunteers played a significant role in promoting continued self-care at home [35]. Older adults with HF already face challenges when using technology; thus, it is not enough to simplify the user interface of the tool, but the technology must be equipped with the proper support channels to meet their needs [36].

One of the key challenges identified by the patients and CPs involved their inability to contact their physician or nurse to help manage their HF symptoms. The inability to communicate with a health care professional led participants to suggest the HFApp to become a source for direct communication to increase their comfort in using the technology and confidence in making self-care decisions. However, as some patients preferred to speak solely to their physician, this became a topic of concern because of barriers associated with physician time constraints [37]. Several studies have found that physicians are less willing to use mHealth tools because of the concern that it will increase their already heavy workload [38]. Assistive technologies, such as mHealth, were initially designed to reduce physician workload; however, if these tools require additional physician involvement, this could threaten the feasibility of the overall intervention [37,39]. In addition, reliance on consistent communication could also become burdensome to patient care, as they have already emphasized their difficulties in adjusting their diuretics with confidence, and any further direct support would decrease their potential to independently make self-care decisions [39]. To prevent an increase in physician workload and patient dependence on provider guidance, we informed patients that physicians and nurses could have access to their HFApp information. Most patients and CPs wanted their physician to be up to date with their condition, as they would not be able to recall all their self-care information during appointments. In reflection of this feedback, we added action items in Multimedia Appendix 3 that would provide mechanisms for voluntary nurse and physician access to patient data and scheduled visits. With this approach, physicians would not be obliged to review their patient data but could access this additional information during appointments. Thus, while recognizing the physician and patient-focused concerns for mHealth tools raised in previous literature, this approach to the HFApp provides an app design that would promote independent self-care and would not limit physicians’ ongoing responsibilities.

As patients and CPs became more comfortable with the idea of using technology for HF self-care, they began to outline specific features they would like to add to customize the app according to their personal preferences and needs. Patients wanted to be able to manage multiple medications for their HF on the HFApp. However, because diuretics are the only medication patients can adjust that have an impact on their weight, we did not include multiple medication management options [40,41]. Other medications for HF management need to be adjusted solely by physicians, advanced practice nurses, or physician assistants as the risk of incorrect dosing can lead to hypotension, bradycardia, or electrolyte imbalances [40,41]. Nevertheless, despite these barriers to patient-led medication adjustments, we found that a recent study has begun to evaluate whether the use of mHealth-enabled telemonitoring program and connected real-time physiological data for clinical decision support and patient self-management can be used to facilitate remote mediation titration (ClinicalTrials.gov Identifier: NCT04205513) [42]. This RCT is currently ongoing; however, its study design highlights the need for further investigation into this topic, to determine the potential opportunity or need to incorporate medication titration within the HFApp [42].

To promote technology use, patients strongly desired the use of notifications. In this context, notifications would be similar to a *nudge* to guide patient behavior. The nudge theory, developed by American economist Richard Thaler, discusses this concept where the nudge serves as a mode of reinforcement or indirect suggestion to promote positive decision making [43,44]. Patients may fail to take their medication or weigh themselves simply because they forget. However, as patients have different schedules and preferences, notifications would need to be tailored to each of the patient’s preferences to increase its effectiveness and prevent the possibility of *nudge fatigue* [44,45]. Consistent with previous studies that have used notifications or reminder systems, it has been reported that alerts or reminders are the most common and effective mode to promote patient self-care behaviors [46,47]. Thus, given that patients used our paper-based SDDST effectively, integrating notifications with adjustable settings within the HFApp should be considered to improve the continued use of the intervention (Multimedia Appendix 3).

Both patients and CPs expressed that the information provided by physicians or nurses was often not understood or not applicable to their condition. The current standard of care involves nurses providing general HF self-care education to patients and CPs during clinic visits and patients being provided standardized HF booklets, based on national guidelines, to take home. However, for patients and CPs to gain the benefits associated with HF education, the information must be simple to understand and specific to the patient [33,48]. Past studies have indicated that individualized education is key to help patients gain the skills needed for adequate self-care, as it accommodates their learning style and level of health literacy [33,49]. In an RCT (n=223) evaluating the effect of a teaching session with a nurse educator, patients were reported to have increased self-care adherence (*P*=.001) and lower risk of rehospitalization (*P*=.02) compared with standard care [50]. In reflection of patient feedback and literature findings, we aim to incorporate a simple and literacy- and numeracy-sensitive HF summary for nurses to educate patients (Multimedia Appendix 3). Building on the theme of usefulness of HF information, a patient also proposed having their electronic medical record (EMR) information connected to the HFApp. This feature or approach would help both patients and physicians, as it would increase the accuracy of their diuretic dosage at home and their diagnosis in the clinic. Nevertheless, as many hospitals utilize various EMR platforms, the compatibility of patient data to the app may be difficult to resolve and the approval for its use may be challenging to obtain. One approach to help mitigate this challenge involves partnering with various health systems or hospitals. Recently, Apple has announced that patients will be able to access their EMR data on their iPhone or iPad because of their partnership with 12 national health systems [51]. They have also partnered with health care software companies such as Epic and Cerner to facilitate interoperability with the app [52]. Thus, with these innovative announcements, the potential for mHealth apps, such as the HFApp, to be seamlessly integrated with EMR systems is becoming more probable.

Toward the end of the persona-scenario discussion, participants had a growing concern regarding the cost of the intervention. They highlighted that continued use of the HFApp could lead to a source of dependency for the intervention, which could jeopardize their health condition if the technology was not covered through health insurance plans. Therefore, a source of long-term funding is needed to confirm patient support. We suggest that by prescribing the HFApp intervention as a treatment or standard of care, we would potentially be able to cover the associated costs through public or private insurance plans [53]. In a qualitative study on oncology providers’ attitude on prescribing mHealth apps, they found that providers were open to recommending or prescribing apps as part of patient care, provided that they have been properly evaluated [54]. Building on this idea, a mixed methods study evaluating the effectiveness of an HF telemonitoring program found that their intervention improved patient self-care and used these results to establish the intervention as part of standard of care at a specialist heart function clinic [55]. The paper-based SDDST has only been evaluated in a pilot study and is not currently being used within standard care; thus, the effectiveness, use, and costs of the HFApp would need to be further investigated to develop the foundational results to support its prescription as standard of care (Multimedia Appendix 3).

Some older adults with HF suggested the integration of the HFApp on their own personal devices (eg, tablet, smartphone, and iPad). They claimed that this could reduce any upfront costs associated with the tool and improve the convenience of the intervention. The HFApp is designed to be used on one device solely for HF self-care, as there is concern that integrating the HFApp on personal devices will potentially complicate the tool and reduce its overall usability [56]. Studies have reported that the additional functions on personal devices create a higher possibility for error or misuse [56]. Thus, it is recommended that for older adults, it may be more beneficial to have one device for one purpose [56].

### Limitations

We aimed to recruit patients with varying self-care adequacy levels to prevent the occurrence of selection bias in our evaluation and to ensure that we would obtain feedback from a range of patients. However, all participants were recruited from the Hamilton General Hospital, which may have created some sampling bias. Throughout the persona-scenario discussion sessions, patients and CPs were also limited in their ability to interact with the HFApp, as we did not have a developed prototype. The focus of this study was to obtain feedback on patient self-care challenges based on the mock-ups and intervention description to inform the design of the HFApp prototype. However, once the HFApp prototype is fully developed, further testing would need to be conducted.

### Future Research

Currently, patients and CPs have reported that one of the greatest issues impeding technology use for HF self-care involves their complex design. Considering this, future studies should look to involve patients with HF and their CPs to inform the design of their intervention. Furthermore, as many other facilitators for mHealth usage involve logistical considerations with nurses and physicians (ie, communication and costs), further investigation into the perspectives of CPs should be completed to evaluate the feasibility of specific tool features.

### Conclusions

To our knowledge, this is the first study that has collected feedback regarding the design of an mHealth app from patients with varying HF self-care adequacy levels. We found that patients with HF were willing to adopt an electronic health app if it was easy to use and customizable to their preferences. Technology has displayed its potential to improve clinical outcomes; however, there is a need to better understand how to improve their adoption among the growing population of older adults. The usability of these tools is strongly dependent on its design; thus, it is important to consult with patients and CPs regarding their needs, challenges, and capabilities to help guide the development of their app.

## Appendix

Multimedia Appendix 1 Patient and informal caregiver persona-scenarios.

Multimedia Appendix 2 Discussion session mock-up designs.

Multimedia Appendix 3 Design theme analysis outlining actions and items to improve the HFApp intervention.

## Abbreviations

CC-SCHFI: caregiver contribution self-care heart failure index
CP: care provider
EMR: electronic medical record
HF: heart failure
mHealth: mobile health
RCT: randomized control trial
SCHFI: self-care heart failure index
SDDST: standardized diuretic decision support tool
UCD: user-centered design
