# Correction: Using an Electronic App to Promote Home-Based Self-Care in Older Patients With Heart Failure: Qualitative Study on Patient and Informal Caregiver Challenges

**DOI:** 10.2196/25624

**Published:** 2020-11-11

**Authors:** Sahr Wali, Karim Keshavjee, Linda Nguyen, Lawrence Mbuagbaw, Catherine Demers

**Affiliations:** 1 Centre for Global eHealth Innovation Techna Institute University Health Network Toronto, ON Canada; 2 Institute of Health Policy, Management and Evaluation Dalla Lana School of Public Health University of Toronto Toronto, ON Canada; 3 InfoClin Toronto, ON Canada; 4 School of Rehabilitation Science Faculty of Health Sciences McMaster University Hamilton, ON Canada; 5 Department of Health Research Methods, Evidence and Impact McMaster University Hamilton, ON Canada; 6 Department of Medicine McMaster University Hamilton, ON Canada

In “Using an Electronic App to Promote Home-Based Self-Care in Older Patients With Heart Failure: Qualitative Study on Patient and Informal Caregiver Challenges” (JMIR Cardio 2020;4(1):e15885) the authors noted two errors.

In the originally published article, Sahr Wali was listed as the Corresponding Author. The Corresponding Author address was listed as:

Institute of Health Policy, Management and Evaluation

Dalla Lana School of Public Health

University of Toronto

155 College St

Toronto, ON, M5T 3M6

Canada

Phone: 1 416 978 4326

Email: sahr.wali@mail.utoronto.ca

In the corrected version, the Corresponding Author has been changed to Catherine Demers, with the Corresponding Author address:

Department of Health Research Methods, Evidence and Impact

McMaster University

237 Barton St E

Hamilton, ON, L8L 2X2

Canada

Phone: 1 905 525 9140 ext 73324

Email: demers@hhsc.ca

An Acknowledgements section has also been added to the original paper with the following text:

This work was completed at McMaster University.

The correction will appear in the online version of the paper on the JMIR Publications website on November 11, 2020, together with the publication of this correction notice. Because this was made after submission to PubMed, PubMed Central, and other full-text repositories, the corrected article has also been resubmitted to those repositories.

