# Supplementary material for: Using an Electronic App to Promote Home-Based Self-Care in Older Patients With Heart Failure: Qualitative Study on Patient and Informal Caregiver Challenges
Source: JMIR Cardio. 2020 Nov 9;4(1):e15885. doi: 10.2196/15885 (PMC7657601; doi:10.2196/15885)
Supplement: Multimedia Appendix 1 [file cardio_v4i1e15885_app1.pdf]

Diane Lambert

Xtensio

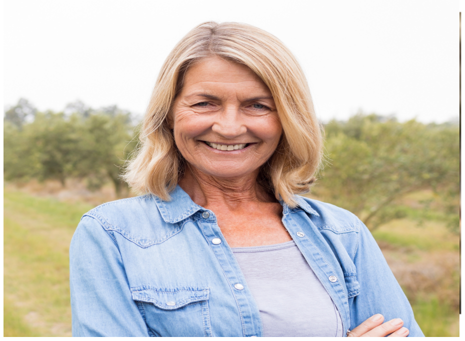

Age: 62
Work: Post Office Worker
Family: Widowed with one daughter
Location: Hamilton, ON
Health: Recently diagnosed with heart failure

Friendly | Forgetful | Dependant on Others

Bio

Diane has lived a healthy lifestyle for the majority of her life. She has worked at the post office for the past 25 years. Her time at the post office has gained her some basic computer skills, but she is still unfamiliar with using new technology like tablets, mobile phones or Ipads.

Diane was not concerned with her health until her recent diagnosis with heart failure (HF). She is now concerned about how she will be able to take care of herself, so she spends her weekends at her daughter's house. Diane would like to be able to keep track of her weight and medication, but she is overwhelmed and tends to forget what she needs to do.

Attitude

Diane gets very emotional about her HF because she feels she is no longer in control. She fears that she will not be able to take care of herself and she will result in getting worse. Her daughter uses her fear to motivate her to use the HF App, so it can help her manage her conditions. Diane is hesitant at first, but she is willing to try for her daughter.

Goals

- Learn how to use HF App without assistance
- Be able to manage her weight and medication on a consistent basis

Frustrations

- Unfamiliar with using Ipads or tablets- She must learn from scratch
- New to self-care regimens - Fears unable to take care of herself
- Diane wants to use HF App but loses faith in herself when she comes across any troubleshooting issues

Scenario	Needs	Behavior
Diane has her scale set up at her house, but on Saturday she takes it with her to her daughters house. She tries to measure her weight the next morning, the scale does not send link to the HF App. The HF App is physically connected to the scale, and Diane had set it up as it was in her home, but she still has trouble measuring her weight.	- Technical Assistance/ Support	Diane opens the HF App to the “Measure my Weight” option and then clicks the “Help” option. She clicks “technical assistance” and then gets connected to a nurse/volunteer via phone. Diane is told to unplug the device, but it still does not work. She ends up having to schedule a visit from the nurse/volunteer to come in to her daughter’s home.

Christina Williams

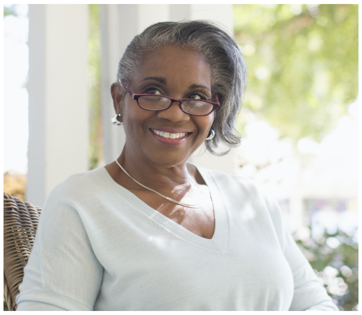

Age: 70
Work: Retired Highschool English Teacher
Family: Married with two sons
Location: Milton, ON
Health: Diagnosed with heart failure >3 years ago

Hardworking

Independent

Creative

Bio

Christina went to the University of Toronto and obtained her Bachelors in English, and went on to become a grade nine English teacher for the past 30 years. She was always felt comfortable learning new things from books to technology. Christina has an Ipad and a computer at home.

Throughout Christina's life, she grew up with both her parents suffering from heart failure (HF). She is well aware of its symptoms and of the self-care procedures, as she took care of both her parents. Christina is comfortable maintaining her own self-care and has done a fairly well good job for the past 3 years, but she has recently noticed that her weight has been inconsistently changing.

Attitude

Christina feels has always been able to be in control of herself, but these recent changes in her weight have led her to seek more help. She came across the HF App through her family doctor, but she does not feel she needs the tool. Christina thinks that the HF App is more for people who forget to take their medication or keep track of their weight, and not for someone like her. She does not feel motivated to use app, but she decides to try it out to see if it can help her with her weight changes.

Goals

- Keep her weight consistent
- Learn more about what contributes to weight changes

Frustrations

- She must keep the tablet and the scale in the same place at all times
- Christina would rather speak to a physician than a nurse/volunteer for follow-up questions

Scenario	Needs	Behavior
Since Christina has used the HF App she has noticed her weight has become more stable. However, this morning her weight had gone up and the HF App recommended a change in her water pill dosage. Christina does not feel confident in the dosage change.	- Additional medication information	Christina opens the HF App to “Take My Medication” and then to the “Why” option. She does not feel the extra information is sufficient, so she gets connected to a nurse/volunteer. She then directly asks to speak to a physician, as she is more comfortable.

Mark Nguyen

Xtensio

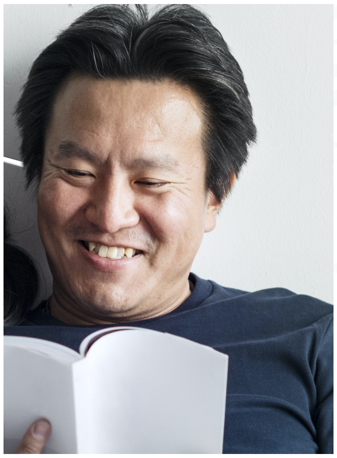

Age: 57
Work: Accountant
Family: Married with one daughter
Location: Burlington, ON

Intelligent | Caring | Helpful

Bio

Mark has worked as an accountant for over 10 years after graduating from McMaster University. Although his career does not fall under the healthcare field, he has become familiar with healthcare teams since his wife was diagnosed with heart failure (HF) two years ago.

Mark recently opened up his own accounting firm, but feels he may have to put his career on hold for his wife. Mark's wife, Cynthia, has constant dizziness and tends to spend a lot of her time sleeping. He is afraid to leave her alone because she may not be able to take care of herself. Cynthia gets along with healthcare workers, but not with family members due to her dementia.

Attitude

Mark does not feel confident in having his wife take care of herself. He has turned to the HF App to see if it can help him regain confidence in his wife's capabilities, but he is doubtful. Mark wants his wife to be independent, but he has to make sure that he has all his questions answered before doing so.

Goals

- Learn HF App works and what services they provide
- Ensure volunteers/nurses provide proper assistance to help patient both in-person and over the phone
- Have his wife be able to manage her symptoms independently

Frustrations

- Mark is not updated on wife's progress/condition in real time
- Only one tablet has HF App connected

Scenario	Needs	Behavior
Mark is at the office and he gets a notification on his phone stating that his wife has not weighed herself this morning.	- Additional information regarding wife's condition	Mark calls his wife but she does not answer the phone. He is overwhelmed because he does not have access to the HF App, so he cannot check if anything serious has occurred. He leaves work and goes home. Mark sees that his wife is simply sleeping. He checks the HF App and everything seems to be in line.

Sarah Murphy

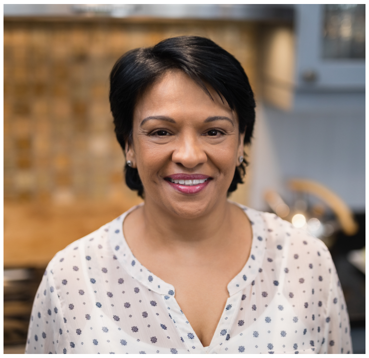

Age: 37
Work: Self-Employed
Family: Married
Location: Toronto, ON

Friendly | Organized | Enthusiastic

Bio

Sarah has a college education in business management. She is self-employed and is looking to open her own ice cream business. Sarah wants to take the next step for her business but does not have a lot of time for her business, because her father has been recently diagnosed with heart failure (HF).

Sarah's father, Jeremy, has had some trouble with his health for the past few years, which has turned Sarah to take on the role as his informal caregiver. She has constant fears for her father. She finds it difficult to communicate with him and she has to consistently remind him about his medications. Sarah is under a lot of pressure at work right now, making it difficult for her to supervise her father.

Attitude

Sarah is struggling between taking care of her father and her business. She is afraid that her father will not be able to take care of himself, and if she is not with him, he will just continue to contact her regardless. She really wants to introduce her father to the HF App. Jeremy is always on his Ipad anyway, so she thinks this will be a great compromise for the both of them.

Goals

- Teach her father how HF App works
- Have her father be able to manage his symptoms independently

Frustrations

- "Help" option contacts nurse/volunteer not Sarah
- Jeremy won't be able to use his Ipad for other applications
  - only HF App runs on device

Scenario	Needs	Behavior
Sarah gets a call from her father and he says that the scale is not working.	- Technical Assistance	Sarah comes home and checks the scale. She finds that the wireless scale is not connecting, so she connects the scale physically with a cable. The scale then connects and her father is able to weigh himself. She asks her father why he didn't contact the volunteer/nurse and he said he didn't know how. She contacts the volunteers and schedules an in-home visit so they can help her father understand how to better use the HF App.
