# Supplementary material for: Using an Electronic App to Promote Home-Based Self-Care in Older Patients With Heart Failure: Qualitative Study on Patient and Informal Caregiver Challenges
Source: JMIR Cardio. 2020 Nov 9;4(1):e15885. doi: 10.2196/15885 (PMC7657601; doi:10.2196/15885)

Welcome to the Heart Success App!

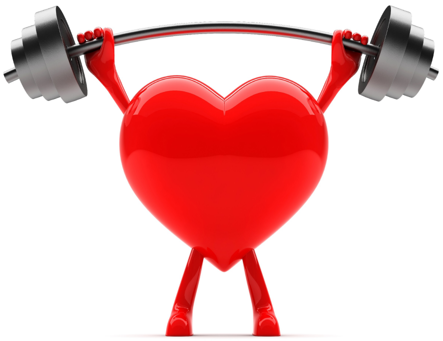

Login **Register**

Hello John Doe & Welcome Back!

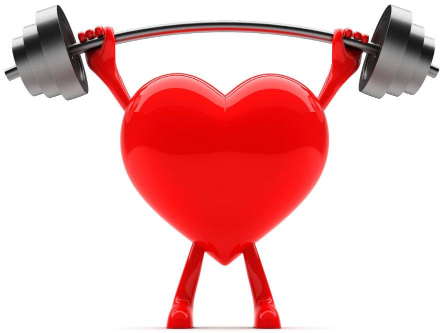

Start

Not You? Logout

Heart Success App Login

Who are YOU?

Patient

Nurse **Volunteer**

Heart Success App Login

User Name:

Password:

Heart Success App Registration

Name:

Patient Profile Username:

Password

CONTINUE

VOLUNTEER

Heart Success App

Please Enter the Patient ID # for your clinical visit

____

CONTINUE

Heart Success App

John Doe’s Ideal Weight: __________

John Doe’s Weight Today: __________

Measure My Weight

Take My Medication

See My History

Measure My Weight

Please Step on the Scale

__________ Kg or Lb

Re-Do **Accept**

Take My Medication

Please Take Your Regular Dose Today

OK **WHY?**

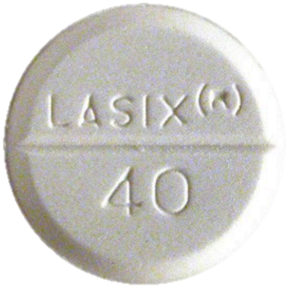

Take My Medication

Please Take ½ Pill From Extra Bottle

OK **WHY?**

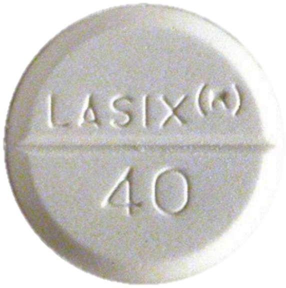

Supplement: Multimedia Appendix 2 [file cardio_v4i1e15885_app2.pdf]
