# Supplementary material for: Using an Electronic App to Promote Home-Based Self-Care in Older Patients With Heart Failure: Qualitative Study on Patient and Informal Caregiver Challenges
Source: JMIR Cardio. 2020 Nov 9;4(1):e15885. doi: 10.2196/15885 (PMC7657601; doi:10.2196/15885)
Supplement: Multimedia Appendix 3 [file cardio_v4i1e15885_app3.docx]

| Factors/design requirement | Quote | Action | Item |
| --- | --- | --- | --- |
| Design theme 1: Usability of technology | | | |
| 1.1. Perception that technology will make self-care more challenging | *“*I don’t like using any technology, it just makes more problems”  *“*I think he would have a lot of difficulty learning how to use it. He barely knows how to use his phone” | - Ensure that patients and CPs^a^ are comfortable with HFApp use. - The nurse provides step-by-step instructions on HFApp use during the initial visit. - Volunteers provide technical assistance (as needed). | - Simple and literacy- and numeracy-sensitive instruction manual on HFApp use for patient and/or CP - Scheduled first nurse visit - Training programs for nurses on HF self-care and HFApp and tablet use - Training programs for volunteers on HFApp and tablet use |
| 1.2. Incentive for technology use not seen | *“*If I’m already writing out my weight everyday and doing fine, I don’t see a reason for me to stop what I’m doing and learn something new, like what’s the point?” | - The nurse outlines the benefits associated with HFApp over long term. - The nurse outlines the simplicity of HFApp and automation of steps. | - Summary for patients on how HFApp reduces self-care challenges (literacy and numeracy sensitive) |
| 1.3. Willingness to use technology if kept simple | “Ya this would be really helpful. I always have to teach my dad how to use things on his phone… that’s smart to add the volunteer part” | - Simplify the user interface of the HFApp. - Provide volunteer assistance (in person and via phone) for technical issues. | - User interface guideline for older adults - Secure app with mechanism to contact volunteers - Volunteer training program - Scheduled volunteer visits |
| Design theme 2: Communication | | | |
| 2.1. Use of direct communication (in person and online) with nurse highly desired | “My doctor even gave me his cell phone number, but I still can’t reach him”  “My weight is not always consistent, so I would want to talk to someone before I change anything” | - Provide direct communication with nurse via the HFApp. - Nurses help with patient self-care using the HFApp. - Volunteers help optimize HFApp use (weight scale calibration and internet connection). | - A secure system to contact nurses - Scheduled nurse and/or volunteer home visits (as needed) |
| 2.2. Open sharing and access to patient information during communication | “The worst is when they think they’re right, but they don’t understand that my symptoms are not the same as before”  “I try to record my weight, but when my cardiologist asks me questions I don’t know what to say...I think having the app track it would be really good for me cause I get lazy” | - Provide nurses and physicians the ability to access patient information on the HFApp. - The physician or the nurse follows regular appointment/call routine in parallel with the content obtained from the HFApp. | - Secure mechanism for nurses and physicians to access the HFApp |
| Design theme 3: App customization | | | |
| 3.1. Management of medications on one device | “I need to track my blood pressure and my water pills. It would be neat if this app could put both of them in it” | - Introduce the HFApp solely for diuretic adjustment. - Patients manage diuretics according to weight. - Nurses determine if patient needs are being met and self-care is being improved during ongoing home visits (via SCHFI^b^). - Determine a suitable duration SCHFI to be conducted over the trial period. | - Tablet - Bluetooth scale - Simple and literacy- and numeracy-sensitive instruction manual on HFApp use for patient and/or CP - Nurses to use SCHFI questionnaire for self-care evaluation - Scheduled SCHFI evaluations |
| 3.2. Addition of notifications at the patient’s desired time/manner | “I always forget to do it. If you don’t tell me I won’t do it. So, if you want me to use this thing, you better buzz me until I do it… For me, I would make sure it kept buzzing me until I got onto that scale” | - Integrate the notification option within the HFApp profile settings. - The nurse sets up notification settings during the first initial visit when explaining the HFApp. - Patient and/or CP can alter notification preferences on the HFApp as needed. | - Mechanism to add and adjust notification settings on the HFApp |
| 3.3. Customization of audio and visual formats for each individual patient during setup | “I know my dad’s vision is getting worse. He’s too stubborn to admit it, but I think maybe if the app could repeat each thing out loud when you click it that would really help. Or just use really big fonts and bright colors”  “I don’t like looking at screens. It hurts my eyes. I only look when it’s dim. My son usually fixes that for me, but maybe the doctor can do it” | - Develop the audio and user interface/screen customization feature (font, color, and contrast adjustment) within the HFApp. - The nurse sets up audio and user interface preferences during the first initial visit during HFApp explanation. - Patient and/or CP can alter audio and user interface according to preferences (as needed). | - Mechanism to add/adjust the audio and visual settings on the HFApp |
| Design theme 4: Complexity of self-care | | | |
| 4.1. Perception that daily management of HF self-care is difficult | “I don’t weigh myself all the time, its just too much work...The only time I pay attention is when I start to lose my breath, but even then I rather see my doctor” | - Determine the self-care concerns specific to each patient and CP. - The nurse provides education to outline the benefits associated with self-care and automation of challenging steps. | - Scheduled discussion of patient and caregiver needs - Summary of results for improved self-care with the HFApp - Simple and literacy- and numeracy-sensitive instructions on HFApp use |
| 4.2. Difficulty with diuretic adjustment | “I weigh myself and if it doesn’t look normal, I still take my regular water pill...I’m afraid something might happen” | - The nurse provides education on SDDST^c^ benefits. - The physician introduces the HFApp as an effective tool during the clinical visit. | - Summary for patients on how the HFApp reduces diuretic adjustment challenges (literacy and numeracy sensitive) |
| 4.3. Benefits of nursing support | “Ya I would love that. Just to come and make sure I’m alright...This beats having to wait for an appointment” | - The nurse organizes the schedule for patient home visits. - The nurse provides patients with self-care support and ensures that the HFApp is used correctly. | - Scheduled nurse home visits - Training program for nurses on HF self-care and HFApp and tablet use |
| Design theme 5: Usefulness of HF-related information | | | |
| 5.1. Provision of information from physicians and nurses difficult to understand | “They keep talking and repeating stuff, but I don’t understand...I just nod my head cause I don’t want to disappoint them” | - The patient and/or the CP is able to access the simplified summary of HF information on the HFApp. - The patient and/or the CP can contact the nurse directly via the HFApp to answer questions. | - Simple and literacy- and numeracy-sensitive summary on general HF-related information - A secure system to contact nurses through the HFApp |
| 5.2. Interest in information relevant to specific patient | “I’m like Christina...what if I could have my doctor use this info on the app when he talks to me. So he has a better idea of what’s going on” | - Integrate patient information on EMR specific for self-care management onto the HFApp. - The HFApp automatically transfers weight and diuretic information onto EMR. - The physician accesses patients’ integrated EMR and HFApp information during patient consult for consolidated review. | - Mechanism for HFApp information to integrate into subset of EMR - Mechanism for the HFApp to be updated with HF-specific information on EMR (diuretic dose) - Secure system to transfer information between HFApp and EMR - Secure mechanism for physicians to access the HFApp during appointment |
| Design theme 6: Long-term use and costs | | | |
| 6.1. Concerns with potential dependence on the HFApp intervention and future costs | “Who’s paying for it? Like this sounds all great and stuff. But how do I know it will be free forever?” | - Create financial guideline/budget for HFApp implementation. - Outline the cost-benefit analysis for intervention implementation. - Determine the primary stakeholder to fund HFApp implementation. | - Report on costs for the HFApp intervention (tablet, Bluetooth scale, and nursing/volunteer support) - Cost-benefit analysis of readmissions/improved self-care versus intervention |
| 6.2. Integration with the current device for long-term use and reduce cost | “If I could connect it to my phone, then I wouldn’t have to have another thing to think about. I think it be easier for me cause I have it with me all the time anyway” | - Introduce the HFApp on a tablet for sole use of self-care. - Determine the effectiveness of the HFApp on a tablet via SCHFI scores across the study period. - Evaluate patients’ willingness to self-care with the HFApp on a tablet alone versus on other devices at the end of the study via PAM.^d^ - Compare patient SCHFI and PAM scores. | - Tablet - Bluetooth scale - Summary of patient SCHFI scores across the study period - Summary of patient PAM scores at the end |

^a^CPs: care providers.

^b^SCHFI: self-care heart failure index.

^C^SDDST: standardized diuretic decision tool.

^d^PAM: patient activation measure.
